# No excess of mitochondrial DNA deletions within muscle in progressive multiple sclerosis

**DOI:** 10.1177/1352458513490547

**Published:** 2013-12

**Authors:** Graham R Campbell, Amy K Reeve, Iryna Ziabreva, Richard Reynolds, Doug M Turnbull, Don J Mahad

**Affiliations:** 1 Centre for Neuroregeneration, University of Edinburgh, Chancellor’s Building, UK; 2 Wellcome Trust Centre for Mitochondrial Research, Newcastle University, UK; 3 Wolfson Neuroscience Laboratories, Imperial College Faculty of Medicine, UK

**Keywords:** Mitochondria, muscle, multiple sclerosis

## Abstract

**Background:**

Mitochondrial dysfunction is an established feature of multiple sclerosis (MS). We recently described high levels of mitochondrial DNA (mtDNA) deletions within respiratory enzyme-deficient (lacking mitochondrial respiratory chain complex IV with intact complex II) neurons and choroid plexus epithelial cells in progressive MS.

**Objectives:**

The objective of this paper is to determine whether respiratory enzyme deficiency and mtDNA deletions in MS were in excess of age-related changes within muscle, which, like neurons, are post-mitotic cells that frequently harbour mtDNA deletions with ageing and in disease.

**Methods:**

In progressive MS cases (*n*=17), known to harbour an excess of mtDNA deletions in the central nervous system (CNS), and controls (*n*=15), we studied muscle (paraspinal) and explored mitochondria in single fibres. Histochemistry, immunohistochemistry, laser microdissection, real-time polymerase chain reaction (PCR), long-range PCR and sequencing were used to resolve the single muscle fibres.

**Results:**

The percentage of respiratory enzyme-deficient muscle fibres, mtDNA deletion level and percentage of muscle fibres harbouring high levels of mtDNA deletions were not significantly different in MS compared with controls.

**Conclusion:**

Our findings do not provide support to the existence of a diffuse mitochondrial abnormality involving multiple systems in MS. Understanding the cause(s) of the CNS mitochondrial dysfunction in progressive MS remains a research priority.

## Introduction

In the majority of patients with multiple sclerosis (MS), neuronal cell bodies and axons degenerate in the context of inflammation and demyelination, leading to a gradual decline in neurological function (progression).^1^ A gathering body of evidence from post-mortem tissue-based studies of progres sive MS and models of MS consistently indicate a role for mitochondria in the dysfunction and degeneration of neurons.^2–5^ However, mitochondria and mitochondrial DNA (mtDNA) have not been investigated in detail in non-central nervous system (CNS) tissue in MS, particularly post-mitotic cells such as skeletal muscle fibres.

Mitochondria are the most efficient producers of energy in the form of ATP. The mitochondrial respiratory chain consists of five complexes, each made up of multiple subunits.^6^ These subunits are encoded by both mtDNA and nuclear DNA. In patients with mtDNA deletions and mtDNA depletion, not all complexes of the mitochondrial respiratory chain are affected; complex IV is frequently impaired while complex II is spared or increased (respiratory enzyme deficiency), as the latter is encoded entirely by the nuclear DNA.^7^ Cells without complex IV activity can be identified in tissue using histochemistry, in contrast to those lacking complex I, for single cell-based studies. We recently identified mtDNA deletions at high levels, arising through clonal expansion, in respiratory enzyme-deficient cortical neurons and choroid plexus epithelial cells in progressive MS, while others described a decrease in transcripts of mitochondrial respiratory chain subunits encoded by nuclear DNA.^2–5,8^ A high level of mtDNA deletions is necessary to impair the biochemical activity of respiratory chain complexes, as each cell contains multiple copies of mtDNA. Clonal expansion of the mtDNA deletion is the process by which an mtDNA deletion reaches high levels in a single cell.^9^ This phenomenon occurs in post-mitotic cells, including neurons and muscle fibres, with ageing and in disease.^10^ If the CNS mtDNA deletions seen in MS were part of a diffuse multisystem process, they would be detectable within respiratory enzyme-deficient skeletal muscle, a key tissue in the diagnosis of mitochondrial disease.^11^

In this study, we investigated single fibres in paraspinal muscles from progressive MS cases, known to harbour an excess of mtDNA deletions in the CNS, to determine whether the respiratory enzyme deficiency and mtDNA deletions were also present in excess of age-related changes within muscle.

## Materials and methods

### Case details

A total of 17 progressive MS cases, where snap-frozen CNS tissue was also available (except MS09), and 15 age-matched controls were used for this study (Table 1). A family history suggestive of a primary mitochondrial disorder was not present and in controls there was no evidence of a neurodegenerative disorder, including Parkinson’s disease (PD). MS cases (except MS09) were non-ambulatory and all the controls remained mobile. All post-mortem muscle (multifidus) from MS and controls were obtained from the United Kingdom (UK) MS and PD tissue bank and stored at −80°C. The peri-operative samples were obtained from lumbar paraspinal and deep cervical paraspinal muscles. Ethical approval was granted for this study (09/H0906/81).

**Table 1.** Clinical details of the multiple sclerosis cases and controls.

Cases	Age (gender)	Tissue type	Type of MS	Disease duration (years)	Time from wheelchair (years)	PM time (hours)
MS01	40 M	PM	SP	23	23	18
MS02	44 F	PM	SP	10	10	9
MS03	45 F	PM	PP	20	13	18
MS04	46 M	PM	SP	20	7	12
MS05	47 F	PM	SP	13	10	27
MS06	49 F	PM	SP	23	11	7
MS07	49 F	PM	SP	17	NA	21
MS08	50 F	PM	SP	NA	NA	22
MS09	51 F	PO	RP	12	0	—
MS10	52 F	PM	SP	15	8	12
MS11	53 M	PM	PP	12	7	15
MS12	54 F	PM	SP	26	6	11
MS13	55 F	PM	SP	27	7	14
MS14	61 M	PM	SP	20	9	24
MS15	72 M	PM	SP	27	NA	23
MS16	75 M	PM	SP	32	16	22
MS17	82 F	PM	SP	45	14	15
CON01	42 F	PO	—	—	—	—
CON02	42 M	PO	—	—	—	—
CON03	51 M	PO	—	—	—	—
CON04	52 F	PO	—	—	—	—
CON05	53 M	PO	—	—	—	—
CON06	57 F	PM	—	—	—	16
CON07	62 F	PM	—	—	—	21
CON18	64 M	PM	—	—	—	18
CON19	64 M	PO	—	—	—	—
CON10	65M	PO	—	—	—	—
CON11	71 M	PM	—	—	—	14
CON12	72 M	PO	—	—	—	—
CON13	74 M	PO	—	—	—	—
CON14	77 M	PM	—	—	—	26
CON15	82 M	PM	—	—	—	21

The mean age of MS cases (54.41 ± 11.63) was not significantly different from controls (61.87 ± 12.22, *p* = 0.09). None of the MS cases were on prescribed disease-modifying therapy. The family history suggestive of a primary mitochondrial disorder was not documented in any of the cases. All MS cases contained mtDNA deletions in excess of age in non-lesion grey matter from either brain or spinal cord (Supplementary Figure 1) and satisfied MacDonald criteria 2001 for the diagnosis of MS.

MS: multiple sclerosis; M: male; F: female; CON: control (as previously described^23^); mtDNA: mitochondrial DNA; NA: not available; PM: post-mortem; PO: peri-operative; PP: primary progressive; RP: relapsing progressive; SP: secondary progressive.

### COX/succinate dehydrogenase (SDH) histochemistry

Cytochrome *c* oxidase or COX (100 µm cytochrome *c*, 4 mM diaminobenzidine tetrahydrochloride and 20 µg/ml catalase in 0.1 M phosphate buffer at pH 7.0) and SDH (130 mM sodium succinate, 200 mM phenazine methosulphate, 1 mM sodium azide, 1.5 mM nitroblue tetrazolium in 0.1 M phosphate buffer pH 7.0) histochemistry was performed on cryostat sections (15 µm thickness) sequentially (COX/SDH) in all cases, as previously stated.^12^

### Immunohistochemistry

Immunohistochemistry was performed on serial cryostat sections to those subjected to histochemistry. Sections were air dried for one hour and fixed in 4% paraformaldehyde (PFA) for 10 minutes. The tissue was then permeabilised in a graded methanol series with additional 0.3% H_2_O_2_. Primary antibodies were applied for 90 minutes and visualised using Menapath X-Cell Plus Polymer detection system (A. Menarini Diagnostics, Wokingham) and DAB chromogen (Supplementary Table 1).

### Laser-microdissection, real-time polymerase chain reaction (PCR), long-range PCR and mtDNA deletion breakpoints

Single respiratory-deficient and respiratory-intact (with complex IV activity) muscle fibres were laser microdissected (Leica). The *MTND1*/*MTND4* assay^13^ was used to establish: 1) the level of mtDNA deletions in single respiratory enzyme-deficient and respiratory enzyme-intact muscle fibres, and 2) wild-type (*MTND1* C_t_ values) and total mtDNA copies (*MTND4* C_t_ values) in the same cell. A standard curve for both *MTND1* and *MTND4* copies was run alongside, in triplicates, as well as control standards where the mtDNA deletion level was known. MtDNA deletion levels from analysed samples were categorised into those with high levels (>60%), and those with low levels (<60%) i.e. the threshold mtDNA deletion level required for a biochemical defect of the respiratory chain. The majority of mtDNA deletions encompass a region including *MTND4* whilst *MTND1* is relatively spared and thus mtDNA deletion levels were calculated through ΔC_t_ (C_tND1–ND4_) using the formula (1−*R*) × 100 where *R*=2^−ΔCt^.^13^

For the purposes of comparing mtDNA copies, membrane slides stained only for SDH were used for laser-microdissection and subsequent real-time PCR, given the interference of mtDNA copy number analysis by COX histochemistry. Sequential sections placed on glass slides allowed for the identification of both respiratory enzyme-deficient and respiratory enzyme-intact muscle fibres on membrane slides. To counter any inter-individual variation of both total and wild-type mtDNA copies, the mean copies of 10 random respiratory enzyme-intact fibres were taken from each case. The copy number from each respiratory enzyme-deficient fibre was then divided by this value to give an mtDNA copy ratio for that case, as previously described.^14^

Long-range PCR was performed using primers covering the majority of the major arc, the primary site of mtDNA deletions, including the common deletion, and the Expand Long Template PCR system (Roche), as previously described.^15^ The DNA products were resolved on a 0.7% agarose gel with ethidium bromide. In addition to muscle, non-lesion CNS grey matter (15 µm × 100 µm × 100 µm) was laser microdissected and subjected to long-range PCR to confirm the excess of mtDNA deletions within the CNS in MS (except MS09, Supplementary Figure 1). The products from long-range PCRs indicating evidence of mtDNA deletions were extracted using a Qiagen gel extraction kit. Sequencing of mtDNA breakpoints was carried out using an ABI 3130xl Genetic Analyser (Applied Biosystems) as previously detailed.^3,15^ Sequences were compared to the revised Cambridge Reference Sequence (rCRS) using Seqscape software.

### Microscopy and quantitation

Quantitation of respiratory-intact and respiratory enzyme-deficient muscle fibre density was carried out using a Zeiss Axioimager 2 at ×20 magnification and shown as a percentage of total muscle fibres. The analysis was performed blinded to disease state, case details and molecular findings (by IZ). The immunohistochemistry profile was achieved by using serial cryostat sections. As serial sections were also used for real-time PCR analysis, it enabled us to determine the immunoreactivity in relation to the molecular profile. Therefore, respiratory enzyme-deficient fibres were categorised into those with high or low level of mtDNA deletions (>60%). Densitometry was carried out on grey-scale images and, to negate any inter-individuality, a ratio was determined for an individual by taking 10 random respiratory intact muscle fibres and used as a standard reference point in that individual. The cross-sectional area as well as the proportion of type I and type II muscle fibres, identified using myosin heavy chain slow and fast, respectively, were assessed by taking images at ×20 magnification and using Image J.

### Statistical analysis

Parametric tests were used for statistical analysis. All statistical comparisons were performed using Graph Pad Prism 4, and *t* test and one-way analysis of variance (ANOVA) were used to compare two or more than two groups, respectively. Post-ANOVA tests were carried out where a *p* value of <0.05 was observed. A *p* value of <0.05 was considered statistically significant.

## Results

### Respiratory enzyme-deficient muscle fibres in MS and controls

**Figure 1.**
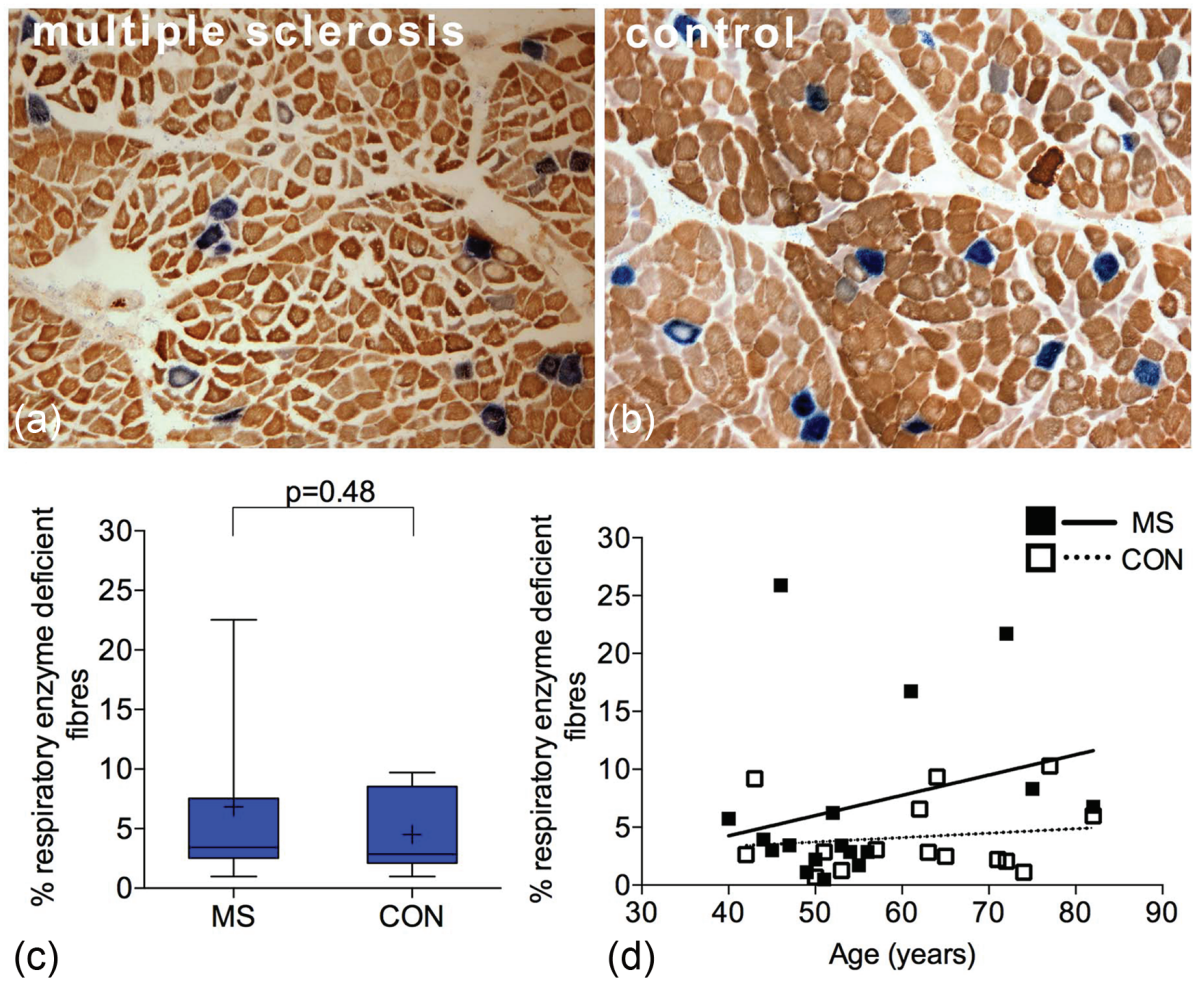
Density of respiratory enzyme-deficient muscle fibres in multiple sclerosis and controls. (a) and (b): Sequential complex IV (COX) and complex II (SDH) histochemistry revealed respiratory enzyme-deficient fibres (stained blue, lacking COX activity with intact SDH) in MS (a) and controls (b). The remaining muscle fibres showed variable intensity of complex IV staining (stained brown). (c): Respiratory enzyme-deficient fibres accounted for a mean of 6.85%±7.44 of all muscle fibres in MS (*n*=17) compared with a mean of 4.51%±3.39 in controls (*n*=15), which was not significantly different (*p*=0.48). (d): There was a positive trend between the density of respiratory enzyme-deficient fibres and age in MS (*p*=0.29, *r*^2^=0.07) and controls (*p*=0.07, *r*^2^=0.32). In MS, this was statistically significant when the three outlying cases (MS04, MS14 and MS15) were excluded (*p*=0.038, *r*^2^=0.312 not shown). COX: cytochrome *c* oxidase; SDH: succinate dehydrogenase; MS: multiple sclerosis. + indicates the mean value. Box plots indicate 25th and 75th centiles with median value by the horizontal line and fifth to 95th centiles by the error bars.

First, we identified respiratory enzyme-deficient muscle fibres using a well-established histochemical technique (COX/SDH histochemistry). Muscle fibres lacking complex IV and with intact complex II are stained blue whereas those fibres with intact complex IV activity are stained brown (Figure 1(a) and (b)). A mean of 1059 muscle fibres were counted per case for both MS and control. The density of respiratory enzyme-deficient fibres in MS (6.85%±7.44) was not significantly different when compared with controls (4.51%±3.39, *p*=0.48) (Figure 1(c)). The mean age of MS cases and controls was comparable (Table 1). There was a positive trend between the density of respiratory-deficient fibres and age in MS and controls, which was not statistically significant (Figure 1(d)). The density of respiratory enzyme-deficient fibres did not correlate significantly with either the disease duration or the duration of relative immobility judged by the time from wheelchair dependence (Supplementary Figure 2(a) and (b)). There were three outlying MS cases with respiratory deficiency exceeding the upper limit of the 95% confidence intervals of controls (MS04, MS14 and MS15).

### Muscle fibre size and type in MS and controls

The cross-sectional area of the respiratory enzyme-deficient muscle fibres did not significantly differ when compared to fibres with intact complex IV activity in both MS cases and controls (*p*=0.059 and *p*=0.258, respectively). However, the cross-sectional area of both type I and type II muscle fibres in MS was significantly lower compared with controls (data not shown). Furthermore, there was a predominance of type I fibres in controls, accounting for 82.30% of all muscle fibres whereas the proportion of type I fibres was significantly decreased to 61.48% in MS (*p*=0.006 not shown).

### Mitochondrial subunit profile of respiratory-deficient muscle fibres in MS

**Figure 2.**
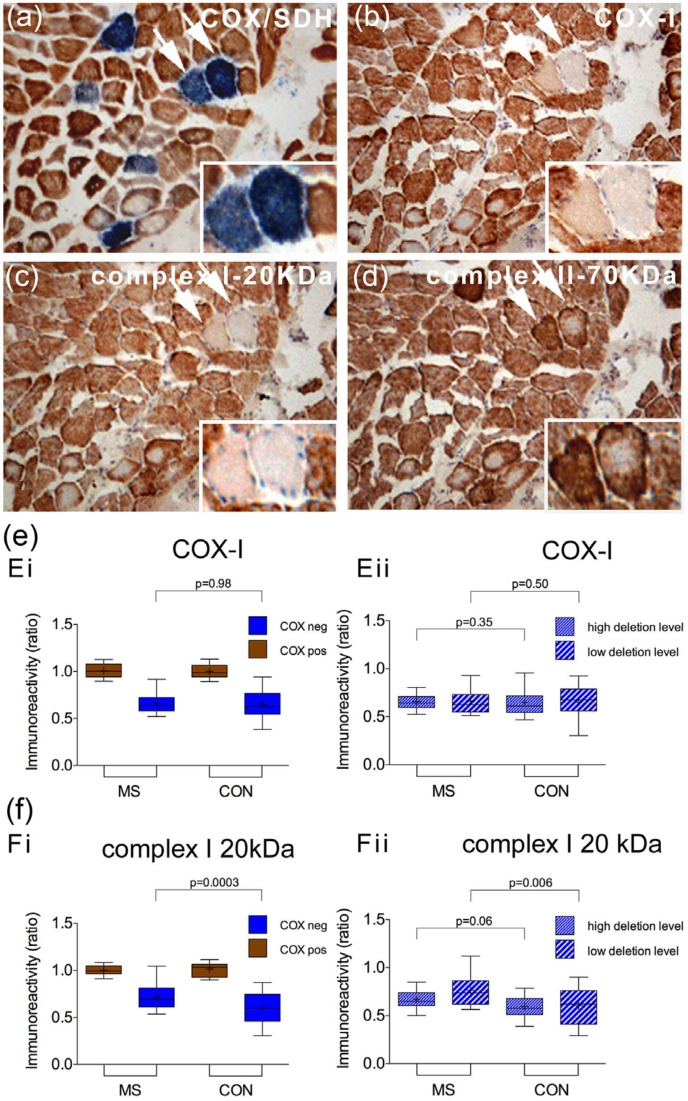
Immunohistochemical profile of mitochondrial respiratory chain subunits in multiple sclerosis muscle. (a–d): In serial sections and in relation to the mitochondrial respiratory chain activity determined by COX/SDH histochemistry (a), the status of mitochondrial DNA (b, complex IV subunit-I) and nuclear DNA ((c) and (d)), complex I 20-KDa and complex II 70-KDa)-encoded mitochondrial subunits was determined using immunohistochemistry in MS cases (a–d) and controls (not shown). In the majority of respiratory enzyme-deficient muscle fibres (stained blue in (a), arrows and insert) the mitochondrial DNA-encoded subunit, COX-I (b), showed low-intensity staining whilst complex II-70kDa staining remained intact or was increased (d). (e) and (f): When single muscle fibres were analysed by densitometry, the loss of both COX-I (Ei, *p*<0.001) and complex I-20kDa (Fi, *p*<0.001) in respiratory enzyme-deficient fibres (stained blue) was found to be significant. When fibres containing high-level (>60%) and low-level mtDNA deletions were analysed separately, we did not detect a significant difference in COX-I (Eii) or complex I 20 kDa (Fii) immunoreactivity between MS cases (*n*=17) and controls (*n*=15). On average 20 fibres were analysed per case for both MS and controls. CON: control; COX: cytochrome *c* oxidase; mtDNA: mitochondrial DNA; Neg: negative or deficiency of complex IV with intact complex II (stained blue); Pos: intact respiratory enzyme activity (stained brown); SDH: succinate dehydrogenase. + indicates the mean value. Box plots indicate 25th and 75th centiles with median value by the horizontal line and fifth to 95th centiles by the error bars.

To assess both mtDNA and nuclear DNA-encoded subunits of the mitochondrial respiratory chain at a single fibre level in MS cases and controls, we tracked the respiratory enzyme-deficient muscle fibres and those with intact complex IV activity in serial sections (Figure 2(a–d)). There was a significant decrease in COX-I (Figure 2(e)) and complex I-20kDa (Figure 2(f)) immunoreactivity in respiratory enzyme-deficient fibres, compared with fibres with intact complex IV activity, in both groups. The extent of subunit loss in respiratory enzyme-deficient fibres in MS, detected by immunohistochemistry, however, was not significantly different compared with controls. This was also the case when the respiratory enzyme-deficient fibres harbouring high- (>60%) and low-level mtDNA deletions were analysed separately in MS and controls.

### MtDNA deletion level in respiratory enzyme-deficient muscle fibres from MS and controls

**Figure 3.**
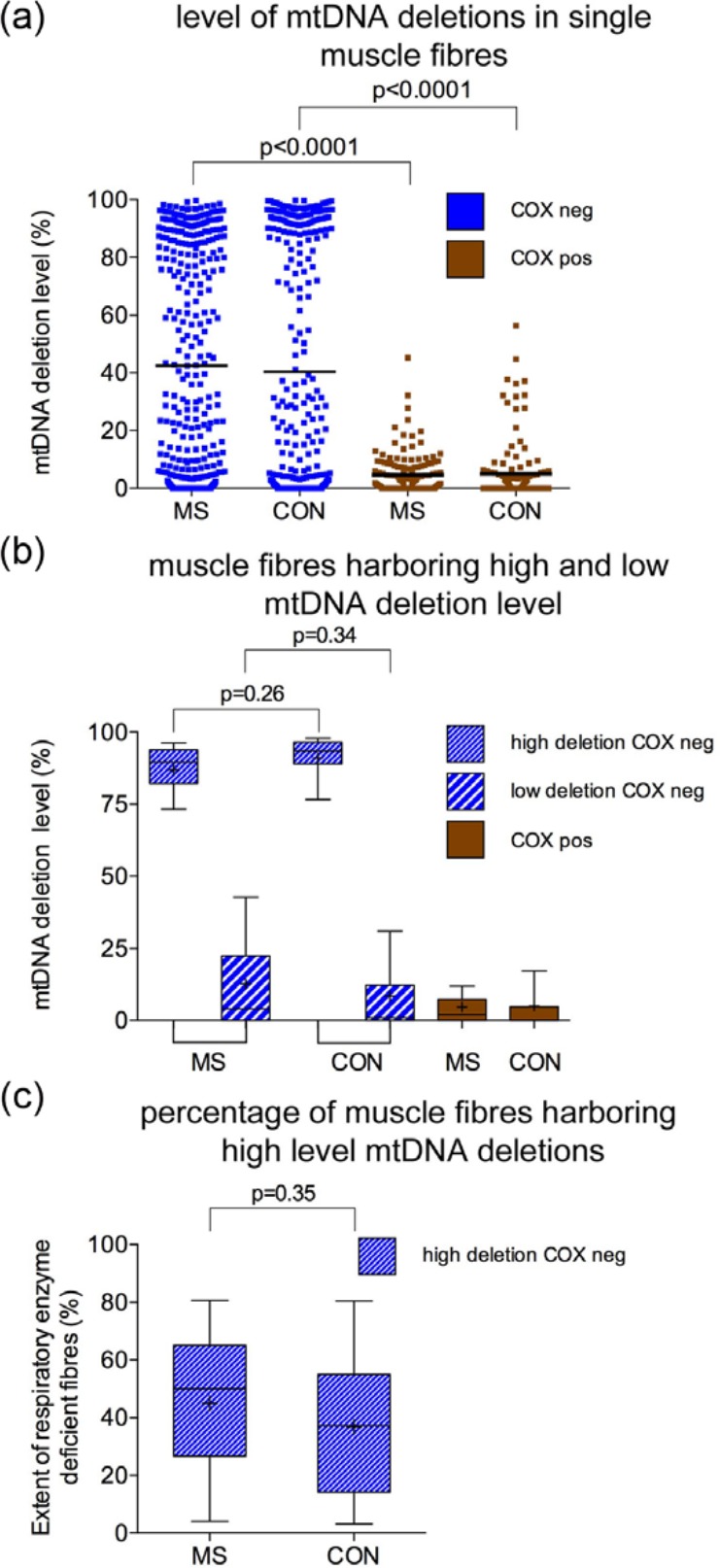
Level of mtDNA deletions in single laser microdissected muscle fibres in multiple sclerosis and controls. (a): Single respiratory enzyme-deficient fibres (COX negative, stained blue) in MS (370 fibres from 17 cases) showed a mean mtDNA deletion level of 45.06% ± 26.35 (a), while the respiratory enzyme-deficient fibres in control (358 fibres from 15 controls) contained a mean mtDNA deletion level of 40.35% ± 42.02, *p*=0.38), which was not significantly different. In both MS and controls, respiratory enzyme-deficient fibres contained significantly greater mtDNA deletion level (*p*<0.0001) than fibres with intact respiratory enzyme activity (stained brown, 4.74% ± 6.93, in MS and 5.06% ± 10.48 in controls). The scatter plots revealed two populations of respiratory enzyme-deficient fibres in both MS and controls. (b): When the respiratory enzyme-deficient fibres (COX negative, stained blue) harbouring high-level mtDNA deletions were considered separately from those containing low-level mtDNA deletions (<60%), the level of mtDNA deletions in neither high- nor low-level fibres was significantly different between MS (*n*=17) and controls (*n*=15). (c): The percentage of respiratory enzyme-deficient fibres containing high level mtDNA deletions (>60%) was determined per case. Although MS cases had a tendency to show a greater percentage than controls (mean of 45.66% in MS and 36.85% in controls), the difference was not statistically significant (*p*=0.35). On average, 20 respiratory enzyme-deficient fibres were analysed per MS case and control by real-time PCR. MS: multiple sclerosis; CON: control; COX: cytochrome *c* oxidase; mtDNA: mitochondrial DNA; Neg: negative or deficiency of complex IV with intact complex II (also termed respiratory enzyme deficient); Pos: intact respiratory enzyme activity; PCR: polymerase chain reaction.

Next, we determined whether the level of mtDNA deletions in single fibres, analysed using real-time PCR and confirmed by long-range PCR and sequencing, and the percentage of fibres harbouring high levels of mtDNA deletions were different between MS and controls (Figure 3). The mean level of mtDNA deletions found within respiratory enzyme-deficient fibres in MS was 45.06% ± 26.35, which was not significantly different compared with controls (40.35% ± 42.02, *p*=0.38). This, however, identified two separate populations of respiratory enzyme-deficient cells: those with above threshold or high level of mtDNA deletions (>60%), sufficient to cause a biochemical defect, and others with a below threshold or low level of mtDNA deletions. When these two populations were analysed separately, the mtDNA deletion levels in fibres with high- or low-level mtDNA deletions were not significantly different in MS cases compared with the corresponding fibres from controls (Figure 3(b)). When MS cases and controls were segregated by age (below and above 60), the difference in mtDNA deletion level between the two groups was not significantly different for <60 (43.95% ± 20.54 versus 39.45% ± 19.42 in MS and controls, respectively, *p*=0.84) and > age 60 (43.55% ± 22.82 versus 39.53% ± 17.93 in MS and controls, respectively, *p*=0.74). As expected, the mtDNA deletion level in all the muscle fibres with intact complex IV activity was <60%. The long-range PCR findings in fibres with high-level mtDNA deletions were consistent with clonal expansion in both MS and controls (Supplementary Figure 3 and Supplementary Table 2). When the percentage of respiratory-deficient fibres harbouring high levels of mtDNA deletions per case was determined, it did not significantly differ between the two groups as a whole (45.66% and 36.85% in MS and controls, respectively, *p*=0.35) or when segregated by age (below and above 60) and it did not correlate with age, disease duration, time from wheelchair bound or the density of respiratory enzyme deficiency (Figure 3(c) and Supplementary Figure 2(c–f)).

**Figure 4.**
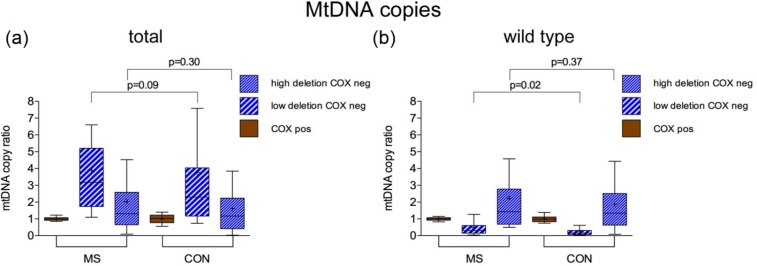
Total and wild-type mtDNA copies in muscle fibres containing low-level mtDNA deletions. (a) and (b): Total mtDNA copies (deleted and wild-type mtDNA copies) were analysed in muscle fibres containing low level (<60%) mtDNA deletions using real-time PCR (a) to determine whether there was mtDNA depletion within these fibres in MS cases. Overall, a significant increase in total mtDNA copies was found in these respiratory enzyme-deficient muscle fibres (in blue) compared to fibres with intact complex IV activity (stained brown) (*p*<0.001), consistent with clonal expansion of mtDNA deletions. The wild-type mtDNA copies were significantly decreased in fibres harbouring high-level mtDNA deletions, as previously recognised, in MS and controls. The extent of wild-type mtDNA depletion in fibres harbouring high-level mtDNA deletions was significantly less (*p*=0.02) in MS compared with controls, despite the difference in ambulation. There was no evidence of a significant decrease in either total or wild-type mtDNA copies within respiratory enzyme-deficient fibres from MS cases compared with controls (b). mtDNA: mitochondrial DNA; PCR: polymerase chain reaction; MS: multiple sclerosis; CON: control; COX: cytochrome *c* oxidase; Neg: negative or deficiency of complex IV with intact complex II (stained blue); Pos: intact respiratory enzyme activity (stained brown). ‘+’ indicates the mean value. Box plots indicate 25th and 75th centiles with median value by the horizontal line and fifth to 95th centiles by the whiskers.

We then determined whether the total mtDNA and wild-type mtDNA copies in respiratory enzyme-deficient muscle fibres were different between MS and controls (Figure 4). As expected, the total mtDNA copies, which includes both mutant and wild-type, was significantly greater while the wild-type mtDNA copies were significantly lower in fibres harbouring high-level mtDNA deletions (*p*<0.0001), indicating clonal expansion of mtDNA deletions in both MS and controls. However, we did not detect a significant decrease in either total or wild-type mtDNA copies within these two subtypes of respiratory enzyme-deficient fibres (those with high- and low-level mtDNA deletions) in MS compared with controls, despite the respiratory enzyme deficiency in these fibres with low level mtDNA deletions and the difference in ambulation between MS cases and controls.

## Discussion

A comprehensive analysis of single muscle fibres did not identify a significant mitochondrial abnormality at the level of respiratory chain activity, expression of subunits of mitochondrial respiratory chain subunits and mtDNA in progressive MS cases. Our findings do not support the existence of a mitochondrial genetic abnormality as part of a diffuse multisystem disorder in cases with classical MS.

The co-existence of an MS-like phenotype with a primary mitochondrial disorder, due to a mutation in mtDNA or nuclear DNA-encoding mitochondrial proteins, has raised the possibility of a diffuse multisystem mitochondrial abnormality in MS. These cases include Harding’s disease, where the MS-like phenotype co-exists with Leber’s Hereditary Optic Neuropathy (LHON), and others where the primary mitochondrial disorder was due to a mutation in the optic atrophy 1 (*OPA1*) gene, mitochondrial tRNA(IIe) gene and mtDNA polymerase gamma (*POLG1*) gene, among others.^16–20^ In our study, two of the three MS cases in which the respiratory enzyme-deficient muscle fibres were prominent had clinical features not usually associated with MS: Gille de la Tourette syndrome (MS04) and retinal vasculitis (MS15). However, these clinical conditions, where mitochondrial dysfunction has been implicated (genetic association studies identified an inner mitochondrial membrane peptidase in Gille de la Tourette syndrome and retinal vasculitis may be a presenting feature of patients with LHON), are not typical or frequent in primary mitochondrial disorders.^21,22^ Although the case reports have suggested the co-existence of a primary mitochondrial disorder with MS as a more frequent occurrence than anticipated by chance, there is no convincing evidence for an aetiological link between pathogenic mitochondrial genetic abnormalities in non-CNS tissue and classical MS, to which our findings also provide support.

Our assessment was limited to paraspinal muscle because ethical considerations prevented the sampling of limb skeletal muscle at post-mortem, which was necessary to procure either brain or spinal cord tissue to confirm the existence of mitochondrial abnormalities in the CNS. We previously reported the age-related mitochondrial changes within paraspinal muscle from both post-mortem cases and patients undergoing spinal surgery.^23^ Our negative findings in MS paraspinal muscles, which can harbour high level of mtDNA deletions in primary mitochondrial disorders,^24^ are supported by a study of open biopsies of quadriceps muscle from MS patients.^25^ Understandably, CNS mitochondria could not be investigated in patients who underwent muscle biopsy. Despite a decrease in complex I activity in muscle, they did not detect mtDNA deletions in the isolated mitochondria. The lack of histochemical techniques to identify complex I-deficient cells hampers the single cell-based analysis of mtDNA, that we and others have used successfully to interrogate complex IV deficient cells. In a separate study of *vastus lateralis* muscle, lactate level, reflecting an underlying mitochondrial dysfunction, was not elevated in the microdialysate from MS patients.^26^ Together, these findings do not support the existence of pathogenic levels of mtDNA deletions in excess of age-related changes within muscle in MS.

The significant morphological changes of muscle fibres (atrophy and fibre-type switching) that we observed in post-mortem tissue from MS cases are most likely related to muscle deconditioning due to the neurological impairment.^27,28^ However, this difference in ambulatory capacity between the two groups is unlikely to have given a false-negative result (mask a mtDNA deletion led mitochondrial abnormality in excess of age in progressive MS cases). Wild-type mtDNA may deplete in disused and deconditioned muscle and such a decrease in wild-type mtDNA copiesin muscle fibres would have caused an apparent increase in the percentage of mtDNA deletions within single fibres in MS (i.e. a false-positive result rather than a false-negative). Our study did not show an apparent loss of mtDNA copies, total or wild-type, in MS cases compared with controls. This may be because continual use of ATP irrespective of ambulatory capacity in the isotonic paraspinal muscles, in contrast to the phasic skeletal limb muscles, minimises the impact of disuse on the mitochondria in paraspinal muscles. Furthermore, rigorous exercise and resistance training was needed to decrease the level of mtDNA deletions in patients with primary mitochondrial disease.^29,30^

Given that mitochondrial abnormalities within the CNS are an established feature of MS, the next challenges in this field are to understand the mechanisms that cause mitochondrial damage in progressive MS, particularly to mtDNA, and determine the consequences of the mitochondrial abnormalities for its pathophysiology. Although mitochondrial abnormalities have been described in experimental autoimmune encephalomyelitis (EAE) and mitochondria have been therapeutically targeted in animal models of MS, mtDNA deletions have not yet been studied in these models.^2,31^ Investigation of mtDNA from the CNS in the existing and new animal models of MS may pave the way to identifying potential novel ways to prevent respiratory enzyme deficiency and identify therapeutic targets for patients with progressive MS.

In summary, a detailed analysis did not identify an excess of respiratory enzyme-deficient fibres, respiratory chain subunit loss or mtDNA deletions in muscle from progressive MS cases. These findings, which do not provide support to the existence of a diffuse mitochondrial abnormality involving multiple systems in MS, highlight the need to investigate the cause(s) of mitochondrial abnormalities, particularly mtDNA deletions, thus far described within neurons in progressive MS.

## References

[1] Dutta R Trapp BD Mechanisms of neuronal dysfunction and degeneration in multiple sclerosis. Prog Neurobiol 2011; 93: 1–12. 20946934 10.1016/j.pneurobio.2010.09.005 PMC3030928

[2] Broadwater L Pandit A Clements R Analysis of the mitochondrial proteome in multiple sclerosis cortex. Biochim Biophys Acta 2011; 1812: 630–641. 21295140 10.1016/j.bbadis.2011.01.012 PMC3074931

[3] Campbell GR Ziabreva I Reeve AK Mitochondrial DNA deletions and neurodegeneration in multiple sclerosis. Ann Neurol 2011; 69: 481–492. 21446022 10.1002/ana.22109 PMC3580047

[4] Dutta R McDonough J Yin X Mitochondrial dysfunction as a cause of axonal degeneration in multiple sclerosis patients. Ann Neurol 2006; 59: 478–489. 16392116 10.1002/ana.20736

[5] Witte ME Nijland PG Drexhage JA Reduced expression of PGC-1alpha partly underlies mitochondrial changes and correlates with neuronal loss in multiple sclerosis cortex. Acta Neuropathol 2013; 125: 231–243. 23073717 10.1007/s00401-012-1052-y

[6] Rahman S Hanna MG Diagnosis and therapy in neuromuscular disorders: Diagnosis and new treatments in mitochondrial diseases. J Neurol Neurosurg Psychiatry 2009; 80: 943–953. 19684231 10.1136/jnnp.2008.158279

[7] Rahman S Lake BD Taanman JW Cytochrome oxidase immunohistochemistry: Clues for genetic mechanisms. Brain 2000; 123 (Pt 3): 591–600. 10686181 10.1093/brain/123.3.591

[8] Campbell GR Kraytsberg Y Krishnan KJ Clonally expanded mitochondrial DNA deletions within the choroid plexus in multiple sclerosis. Acta Neuropathol 2012; 124: 209–220. 22688405 10.1007/s00401-012-1001-9 PMC3674417

[9] Campbell GR Mahad DJ Clonal expansion of mitochondrial DNA deletions and the progression of multiple sclerosis. CNS Neurol Disord Drug Targets 2012; 11: 589–597. 22583438 10.2174/187152712801661194

[10] Larsson NG Somatic mitochondrial DNA mutations in mammalian aging. Annu Rev Biochem 2010; 79: 683–706. 20350166 10.1146/annurev-biochem-060408-093701

[11] McFarland R Taylor RW Turnbull DM A neurological perspective on mitochondrial disease. Lancet Neurol 2010; 9: 829–840. 20650404 10.1016/S1474-4422(10)70116-2

[12] Johnson MA Bindoff LA Turnbull DM Cytochrome c oxidase activity in single muscle fibers: Assay techniques and diagnostic applications. Ann Neurol 1993; 33: 28–35. 8388186 10.1002/ana.410330106

[13] Krishnan KJ Bender A Taylor RW A multiplex real-time PCR method to detect and quantify mitochondrial DNA deletions in individual cells. Anal Biochem 2007; 370: 127–129. 17662684 10.1016/j.ab.2007.06.024

[14] Yu-Wai-Man P Lai-Cheong J Borthwick GM Somatic mitochondrial DNA deletions accumulate to high levels in aging human extraocular muscles. Invest Ophthalmol Vis Sci 2010; 51: 3347–3353. 20164450 10.1167/iovs.09-4660 PMC2904001

[15] Reeve AK Krishnan KJ Elson JL Nature of mitochondrial DNA deletions in substantia nigra neurons. Am J Hum Genet 2008; 82: 228–235. 18179904 10.1016/j.ajhg.2007.09.018 PMC2253975

[16] Harding AE Sweeney MG Miller DH Occurrence of a multiple sclerosis-like illness in women who have a Leber’s hereditary optic neuropathy mitochondrial DNA mutation. Brain 1992; 115 (Pt 4): 979–989. 1393514 10.1093/brain/115.4.979

[17] Horvath R Hudson G Ferrari G Phenotypic spectrum associated with mutations of the mitochondrial polymerase gamma gene. Brain 2006; 129: 1674–1684. 16621917 10.1093/brain/awl088

[18] Palace J Multiple sclerosis associated with Leber’s Hereditary Optic Neuropathy. J Neurol Sci 2009; 286: 24–27. 19800080 10.1016/j.jns.2009.09.009

[19] Taylor RW Chinnery PF Bates MJ A novel mitochondrial DNA point mutation in the tRNA(Ile) gene: Studies in a patient presenting with chronic progressive external ophthalmoplegia and multiple sclerosis. Biochem Biophys Res Commun 1998; 243: 47–51. 9473477 10.1006/bbrc.1997.8055

[20] Verny C Loiseau D Scherer C Multiple sclerosis-like disorder in OPA1-related autosomal dominant optic atrophy. Neurology 2008; 70: 1152–1153. 18287570 10.1212/01.wnl.0000289194.89359.a1

[21] Nociti V Fasano A Bentivoglio AR Tourettism in multiple sclerosis: A case report. J Neurol Sci 2009; 287: 288–290. 19695578 10.1016/j.jns.2009.07.009

[22] Patte M Rouher FN Vernay D Proliferative retinal vasculitis and multiple sclerosis: A case report [in French]. J Fr Ophtalmol 2003; 26: 381–385. 12843896

[23] Campbell GR Reeve A Ziabreva I Mitochondrial DNA deletions and depletion within paraspinal muscles. Neuropathol Appl Neurobiol 2013; 39: 377–389. 22762368 10.1111/j.1365-2990.2012.01290.x PMC4063338

[24] Sakiyama Y Okamoto Y Higuchi I A new phenotype of mitochondrial disease characterized by familial late-onset predominant axial myopathy and encephalopathy. Acta Neuropathol 2011; 121: 775–783. 21424749 10.1007/s00401-011-0818-y PMC3098999

[25] Kumleh HH Riazi GH Houshmand M Complex I deficiency in Persian multiple sclerosis patients. J Neurol Sci 2006; 243: 65–69. 16413582 10.1016/j.jns.2005.11.030

[26] Mahler A Steiniger J Bock M Is metabolic flexibility altered in multiple sclerosis patients? PloS One 2012; 7: e43675. 22952735 10.1371/journal.pone.0043675 PMC3429505

[27] Abadi A Glover EI Isfort RJ Limb immobilization induces a coordinate down-regulation of mitochondrial and other metabolic pathways in men and women. PloS One 2009; 4: e6518. 19654872 10.1371/journal.pone.0006518 PMC2716517

[28] Brierley EJ Johnson MA James OF Effects of physical activity and age on mitochondrial function. QJM 1996; 89: 251–258. 8733511 10.1093/qjmed/89.4.251

[29] Murphy JL Blakely EL Schaefer AM Resistance training in patients with single, large-scale deletions of mitochondrial DNA. Brain 2008; 131: 2832–2840. 18984605 10.1093/brain/awn252

[30] Parise G Brose AN Tarnopolsky MA Resistance exercise training decreases oxidative damage to DNA and increases cytochrome oxidase activity in older adults. Exp Gerontol 2005; 40: 173–180. 15763394 10.1016/j.exger.2004.09.002

[31] Witte ME Geurts JJ de Vries HE Mitochondrial dysfunction: A potential link between neuroinflammation and neurodegeneration? Mitochondrion 2010; 10: 411–418. 20573557 10.1016/j.mito.2010.05.014

